# Early and Midterm Outcomes of “No-Touch” Saphenous Vein Grafts in Japanese Institutions

**DOI:** 10.21470/1678-9741-2022-0121

**Published:** 2022

**Authors:** Hiroshi Tsuneyoshi, Shuji Setozaki, Hideyuki Katayama, Takuki Wada, Shuntaro Shimomura, Akira Takeuchi, Atsushi Sugaya, Tatsuhiko Komiya

**Affiliations:** 1 Department of Cardiovascular Surgery, Shizuoka General Hospital, Kurashiki, Kayama, Japan; 2 Department of Cardiovascular Surgery, Kurashiki Central Hospital, Kurashiki, Kayama, Japan

**Keywords:** Coronary Artery Bypass, Saphenous Vein, Arterial Pressure, Cardiac Catheterization, General Surgery, Peripheral Nervous System Diseases.

## Abstract

**Introduction:**

There have been several attempts to overcome the poor graft patency of saphenous vein grafts. “No-touch” saphenous vein graft (NT-SVG) could be a solution to improve graft patency. We aimed to investigate the early and midterm outcomes of coronary artery bypass grafting (CABG) using NT-SVGs in our hospitals.

**Methods:**

This is a retrospective study of 105 patients who underwent CABG using 130 NT-SVGs between August 2013 and December 2021. NT-SVGs were harvested with about a 5-mm margin of surrounding tissue on both sides of the vein with minimal manipulation. Then, the NT-SVG was dilated by natural arterial pressure without manual distension. After surgery, most of NT-SVGs were assessed by cardiac catheterization or multidetector computed tomography (MDCT) to determine early graft patency. Late graft assessments by MDCT were performed about every five years after surgery.

**Results:**

The early graft patency of NT-SVGs was 100% (125/125); however, two cases of graft twisting were found. Both cases spontaneously resolved. Leg wound infections of NT-SVG harvesting site were seen in 6.2% of patients. Peripheral neuropathy of the legs such as skin numbness and tingling were frequently observed, which lasted up to one year, but no more than two years after surgery. The midterm graft patency of NT-SVGs was excellent (five-year patency of NT-SVGs was 95.8%).

**Conclusion:**

The early and midterm graft patency of NT-SVGs was satisfactory. Although leg wound complications can be seen on the harvesting NT-SVG site, the “no-touch” harvesting technique of SVG could improve graft patency and clinical outcomes of CABG.

**Table:** 

Abbreviations, Acronyms & Symbols	
CABG	= Coronary artery bypass grafting
MDCT	= Multidetector computed tomography
NT-SVG	= “No-touch” saphenous vein graft
SVGs	= Saphenous vein grafts
PVI	= Pulmonary vein isolation
TTFM	= Transit-time flow measurement

## INTRODUCTION

Although saphenous vein grafts (SVGs) are the most frequently used bypass conduits in coronary artery bypass grafting (CABG), the downside of the vein grafts is poor graft patency in both the early and late phases. Past clinical trials^[^1^,^2^]^ revealed that the patency of vein grafts is inferior to that of arterial grafts. Even within one year after surgery, postoperative graft assessments showed vein graft occlusion of about 10% to 20%^[^3^,^4^]^. Conversely, no-touch SVGs (NT-SVGs) that were harvested with pedicle tissue and without high-pressure saline distension provided promising graft patency. Samano et al.^[^5^]^ presented that NT-SVGs achieved 83% of graft patency at a mean time of 16 years after surgery. Recently, a large randomized multicenter trial in China^[^6^]^ was conducted to evaluate the outcomes of NT-SVGs. This trial included 2,655 patients to be randomized 1:1 between NT-SVGs and conventional harvested saphenous grafts and showed a significantly lower graft occlusion rate of NT-SVGs at 12 months after surgery (3.7% *vs.* 6.5%, NT-SVGs *vs.* conventional SVGs, respectively). Today, NT-SVG is recognized worldwide as an improved method for graft patency of SVGs. This harvesting technique is a IIb recommendation in the European guidelines^[^7^]^ and is becoming increasingly popular in Japan^[^8^]^. We have been using NT-SVGs in CABGs since 2013. In this article, we present our early and midterm clinical outcomes of CABGs using NT-SVGs.

## METHODS

We started using NT-SVGs in Kurashiki Central Hospital in August 2013 and then in Shizuoka General Hospital in April 2017. This study included isolated and concomitant CABG cases using NT-SVGs performed by a single surgeon (H.T.) who learned the NT-SVG harvesting technique as a clinical fellow at Sunnybrook Health Sciences Center in Toronto, Canada. The isolated CABG was performed mostly off-pump at both institutions. Concomitant procedures included the aortic valve, mitral valve, maze, and aortic surgeries.

NT-SVGs were harvested in an open manner either by a senior fellow or a resident supervised by an attending surgeon. In the operating room, the patient’s legs were scanned by ultrasound so the surgeon could place markings along the saphenous veins. NT-SVGs were harvested mainly from the lower legs; however, if the diameter of a saphenous vein was < 2 mm in the lower legs, we harvested the NT-SVG from the thigh. Multiple skip incisions were made along the vein markings. Then the saphenous vein was harvested using electrocautery with about a 5-mm margin of surrounding tissue on both sides of the vein. All major side branches were ligated with 4-0 silk or by metal clip. The vein was pulled from its basal bed using scissors. Before removal of the vein, the vein’s surface was marked with a pen to prevent its twisting. Then a small adaptor was inserted into the distal end of the vein and secured with a ligature. After general heparinization, the NT-SVG was connected to the 4F sheath inserted into the femoral artery to let it dilate by natural arterial pressure in off-pump CABG. In case of on-pump CABG or concomitant CABG using cardiopulmonary machine, the NT-SVG was connected to the side branch of the arterial cannula inserted into the ascending aorta. Manual distension of the vein using a saline syringe was strictly prohibited in NT-SVGs. Before closing the skin, a 10F Blake drain was inserted into the vein-harvesting site. The leg wounds were closed in two layers: interrupted 3-0 sutures to the subcutaneous tissue and a continuous 4-0 subcuticular suture. An elasticated bandage was applied to the leg wound during the surgery and kept in place for one week. A 10F Blake drain was removed when total drainage per day was < 10 mL.

In off-pump CABG, the left anterior descending coronary artery was first revascularized by using an *in-situ* internal thoracic artery graft while leaving the NT-SVG to be dilated spontaneously by the natural arterial pressure from the femoral sheath. The NT-SVG was anastomosed to either the left circumflex coronary artery territory or the right coronary artery territory, or both. A sequential anastomotic technique for NT-SVG was used in some cases. Then, the proximal side of the NT-SVG was anastomosed to the ascending aorta. In all cases, an intraoperative assessment of the ascending aorta was performed using epiaortic echography. If patients did not have an atherosclerotic change on the ascending aorta, a partial clamp was performed, and the proximal part of the NT-SVG was anastomosed after creating a 4.0-mm circular punch defect. In case of on-pump CABG or concomitant CABG using cardiopulmonary machine, the distal anastomosis of the NT-SVG was performed under cardiopulmonary machine. After the valve or aortic surgery was finished, the proximal side of the NT-SVG was anastomosed to the ascending aorta. During the operation, all NT-SVGs were examined using transit-time flow measurement (TTFM). As postoperative management, intravenous nitroglycerin was administered for about 24 hours. All the patients undergoing isolated CABG received 100 mg of aspirin and 75 mg of clopidogrel postoperatively. If patients needed blood-thinning agent in case of concomitant surgery, warfarin was used instead of clopidogrel.

After surgery, most patients underwent graft assessments by cardiac catheterization or multidetector computed tomography (MDCT) coronary angiograms within the same hospitalization as part of a routine postoperative study. The NT-SVGs were categorized as either patent or occlusions. The NT-SVGs not visualized during angiographic assessment were defined as occlusions. For sequential anastomosis of NT-SVGs, one occlusion of any of the distal anastomoses was judged as an occlusion of the whole graft. Interventional cardiologists at both institutions independently reviewed the results of cardiac catheterization or MDCT coronary angiograms. Perioperative complications were assessed, which included hospital death, stroke, myocardial infarction, reoperation for bleeding, and mediastinitis. Stroke was defined as a central neurologic deficit persisting for more than 24 hours or new infarcted lesion detected by head computed tomography scan. Myocardial infarction was diagnosed when the electrocardiogram showed new Q-wave or loss of R-wave progression or when a creatine kinase myocardial band enzyme of > 10% was found.

Patients were followed up in the outpatient clinic at postoperative three and 12 months, and then every year. The follow-up rate was 96%. If patients experienced a recurrence of angina or other abnormal results of exercise electrocardiographic testing, late graft assessments were performed using cardiac catheterization or MDCT coronary angiograms. Regardless of whether patients had any symptoms, late graft assessments by MDCT were performed about every five years after the surgery as part of a routine checkup. Of the patients in this study, 50% underwent late graft assessments (> 6 months after surgery).

Leg wound healing and symptoms of the NT-SVG harvesting site were assessed during hospitalization and in the outpatient clinic after discharge. Patients were queried for leg discomfort such as tingling pain or skin numbness at the SVG harvesting site. Leg wound infections included delayed wound healing and the situation when patients needed wound debridement, re-suturing, or negative pressure wound therapy.

### Statistical Analysis

Estimated survival rates and late graft patency of NT-SVGs were calculated using the Kaplan-Meier method with EZR^[^9^]^ (Saitama Medical Center, Jichi Medical University, Saitama, Japan), which is a graphical user interface for R (The R Foundation for Statistical Computing, Vienna, Austria). More precisely, it is a modified version of R commander designed to add statistical functions frequently used in biostatistics.

## RESULTS

**Fig. 1:**
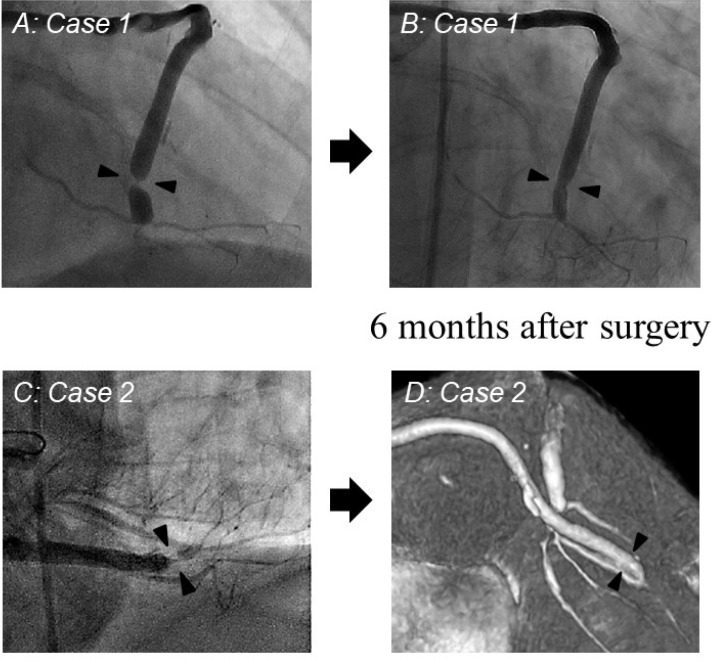
Contrast angiography one week after surgery demonstrating graft twisting (black arrows: A and C) in “no-touch” saphenous vein grafts. At six months after surgery, spontaneous resolution of graft twisting was observed in contrast angiography and enhanced computed tomography angiogram (black arrows: B and D).

We used 130 NT-SVGs in 105 isolated and concomitant CABG cases between August 2013 and December 2021. Table 1 shows the patients’ profiles in this study. Table 2 shows the operative characteristics. Eighty-six percent of cases were isolated CABGs. The number of bypass grafts per patient was 3.4±0.8, including 1.6±0.4 of arterial grafts. Among 130 NT-SVG cases, 33% of NT-SVGs were used as a sequential graft. Percentages of target coronary artery for NT-SVGs was 69% in the right coronary artery, 21% in the left circumflex, and 10% in diagonal branch. We did not use NT-SVGs for left descending artery bypass. The early outcomes of CAGB using NT-SVGs are shown in Table 3. Early graft patency of NT-SVGs was assessed in 125 grafts (96%), which revealed that the patency rate was 100% (125/125); however, two cases (1.6%:2/125) presented graft twisting of NT-SVG (Figure 1). Both patients did not present any chest symptoms or ischemia; therefore, we elected not to perform any catheter intervention on the twisted NT-SVGs, and we gave them warfarin (International Normalized Ratio was controlled about 1.5-2.0) in addition to aspirin to prevent vein graft occlusion. At six months after surgery, both patients underwent reassessments of the grafts, which revealed no evidence of any graft twisting in the NT-SVG. Spontaneous resolution of graft twisting in both NT-SVGs was observed (Figure 1). There were no hospital deaths; two patients had perioperative strokes, one patient had a myocardial infarction, no patient required reoperation for bleeding, and two patients had mediastinitis. Leg wound infections were seen in eight patients (6.2%). Follow-up of leg discomforts in the harvesting site are shown in Table 4.

**Table 1:** Patients’ profiles.

Age (years)	66.1±9.4
Male (%)	89%
Diabetes mellitus	38%
Hypertension	84%
Hyperlipidemia	73%
Current smoker	7%
Hemodialysis	9%
Previous myocardial infarction	22%
Previous stroke	16%
Peripheral arterial disease	12%
Low ejection fraction (< 40%)	8%

**Table 2:** Operative data.

	CABG n=105
Isolated CABG, n (%)	86 (82%)
Off-pump in isolated CABG, n (%)	81 (94%)
On-pump in isolated CABG, n (%)	5 (6%)
Concomitant CABG, n (%)	19 (18%)
Valve surgery, n	18
Arrythmia surgery (maze, PVI), n	2
Aortic surgery, n	1
Anastomoses per patient, n	3.4±0.8
Arterial grafts per patient, n	1.6±0.4
NT-SVGs, n	130
Sequential grafts in NT-SVGs, n (%)	43 (33%)
Target coronary artery of NT-SVG	
Right coronary artery, (%)	69%
Left circumflex, (%)	21%
Diagonal, (%)	10%
Left descending artery, (%)	0%

CABG=coronary artery bypass grafting; NT-SVG=“no-touch” saphenous vein graft; PVI=pulmonary vein isolation

**Table 3:** Early outcomes of CABG using NT-SVGs.

	No. of NT-SVGs n=130
Early graft patency of NT-SVGs, n (%)	125/125 (100%)
Twisted grafts of NT-SVGs, n (%)	2/125 (1.6%)
Hospital death, n (%)	0 (0%)
Stroke, n (%)	2 (1.9%)
Myocardial infarction, n (%)	1 (1%)
Reoperation for bleeding, n (%)	0 (0%)
Mediastinitis, n (%)	2 (1.9%)
Leg wound infection, n (%)	8 (6.2%)

CABG=coronary artery bypass grafting; NT-SVG=“no-touch” saphenous vein graft

**Table 4:** Follow-up of leg discomforts in the harvesting site.

	Just after surgery	3 months after surgery	1 year after surgery	2 years after surgery
Leg discomforts, n (%)	93/101	50/95	9/80	0/52
Tingling or skin numbness, (%)	92%	53%	11%	0%

**Fig. 2:**
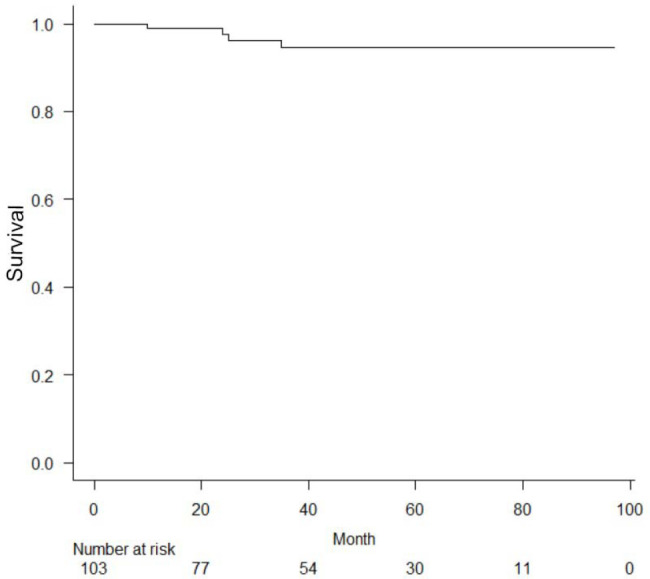
Survival free from overall death.

**Fig. 3:**
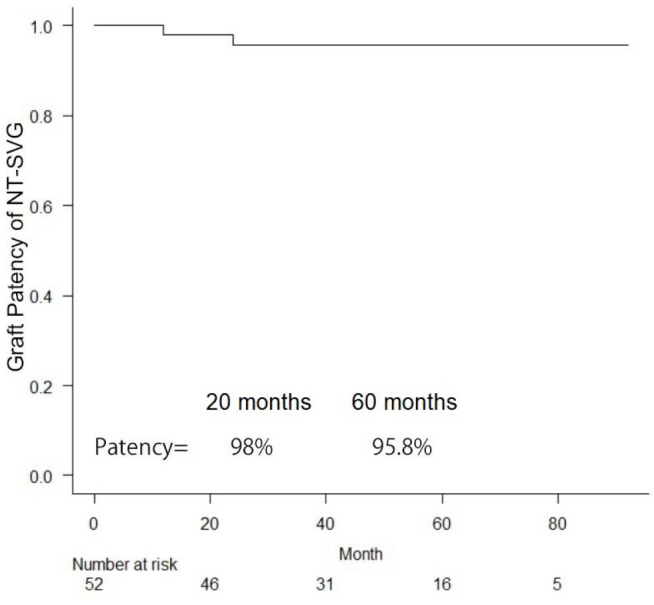
Midterm graft patency of “no-touch” saphenous vein grafts (NT-SVGs).

The mean follow-up duration of the present study was 43 months. The Kaplan-Meier cumulative survival of CABG using NT-SVGs is shown in Figure 2. The midterm graft patency of NT-SVGs is shown in Figure 3 (the five-year patency of NT-SVGs was 95.8%).

## DISCUSSION

In historical studies^[^1^,^2^]^, an arterial graft has been documented to improve long-term graft patency in CABGs. However, SVG is still an important and useful bypass conduit for coronary revascularization because of its easy handling, quick availability, and customizable length. In fact, SVGs are used worldwide in > 80% of CABG cases^[^10^]^ in Japan too. We cannot deny the use of SVGs even in the present era when arterial grafts are recommended to be used for CABG. Of course, disadvantages of the vein graft include lower graft patency as compared with arterial grafts. Several attempts have been introduced to overcome the poor graft patency of SVGs - pharmacotherapy^[^11^]^, buffered storage solutions^[^12^]^, gene therapy^[^13^]^, external stenting^[^14^]^, and harvesting technique^[^8^]^. NT-SVG is one of the solutions to improve graft patency. In this study, we have also shown good early and midterm clinical outcomes of NT-SVGs. The early graft patency was 100% (125/125) and midterm graft patency was 96%.

There are several possible explanations for the protective effect of NT-SVGs against graft occlusion. NT-SVGs could be associated with better endothelial function than conventionally harvested SVGs^[^15^]^. The vasodilatation and vasoconstriction responses are better preserved in NT-SVGs^[^16^]^, which could be beneficial in preventing graft occlusion. NT-SVGs could keep more vasa vasorum^[^17^]^, which supply nutrients and oxygen to the vein wall, rather than conventionally harvested SVGs. Intact vasa vasorum of the surrounding tissue can keep the vein alive and prevent graft failure.

A major drawback of the NT-SVG technique is a higher rate of wound infections in legs^[^18^]^ because the tissue defect under the leg skin at the harvest site is large. Skin flaps could delay wound healing. Moreover, peripheral neuropathies, such as skin numbness and tingling, were observed in the legs, which may result from nerve injury near the SVG. Small neuron fibers close to the SVG could be harvested with surrounding tissue of NT-SVGs; however, in the present study, these peripheral symptoms were observed up to one year, but no more than two years after surgery, which may not impair a patient’s quality of life. If a major saphenous nerve near the SVG can be preserved during NT-SVG harvesting, peripheral neuropathy could be reduced. In contrast, leg wound infection can impair a patient’s quality of life and prolong their hospital stay. In this study, after harvesting NT-SVGs, 6.2% of patients had leg wound infections that needed surgical intervention. This occurrence was lower than that of previous report of NT-SVGs in the SUPERIOR SVG study^[^18^]^, which presented that 23% of patients suffered from harvesting site infections. At our institutions, leg wounds were carefully closed with interrupted subcutaneous sutures and continuous subcuticular sutures. An interrupted subcutaneous suture can prevent fat necrosis under the skin unlike a continuous suture. Moreover, a leg wound drain tube was usually placed for few days until the amount of drainage significantly decreased. This meticulous wound management could reduce leg wound complications after harvesting NT-SVGs.

**Fig. 4:**
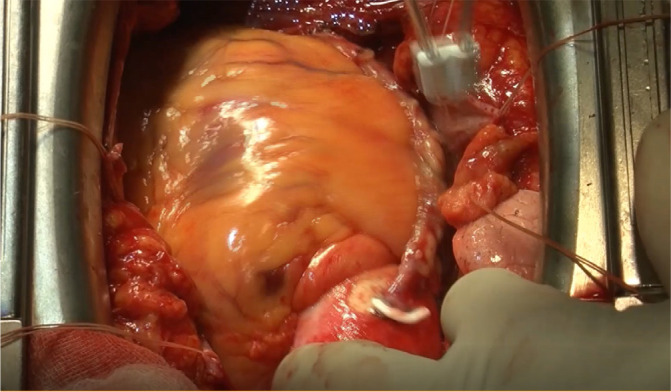
“No-touch” saphenous vein graft was attached to the epicardium using fibrin glue.

One pitfall of NT-SVGs is vein twisting. Surround tissue of NT-SVGs may disturb us to find the graft twisting during surgery; therefore, vein marking on the top of the NT-SVG immediately after harvesting is important to prevent twisting. However, we experienced NT-SVG twisting in two cases, even though twisted NT-SVGs were marked properly and checked by the graft flow using a TTFM. The graft flow of NT-SVGs did not show any problem after the completion of graft anastomosis. In both cases, the twisted site of NT-SVGs in postoperative imaging was close to the distal anastomoses. The slight torsion of NT-SVGs may gradually advance toward the distal part of the vein after surgery and could cause severe stenosis at the distal anastomosis site. After we experienced two twisted NT-SVGs, all NT-SVGs were routinely attached on the epicardium using fibrin glue (Figure 4). At our institutions, this surgical maneuver has prevented any further experiences of NT-SVG twisting. In general, SVGs are vulnerable to graft twisting, which attenuates graft flow and commonly results in graft occlusion. Although severe tortuosity occurred in NT-SVGs, two twisted NT-SVGs remained open, which could show the superiority of NT-SVGs over conventionally harvested SVGs.

### Limitations

This study had several limitations. First, this was a retrospective study with a small number of patients in limited institutions. Second, this study did not compare the results of conventionally harvested SVGs. Third, the follow-up of this study was only up to the midterm period. Long-term results are necessary to determine the benefits of NT-SVGs. Further studies of long-term outcomes in larger numbers of patients using NT-SVGs are warranted.

## CONCLUSION

In our institutions, early and midterm graft patency of NT-SVGs was satisfactory. Moreover, because of our meticulous wound management, the occurrence of leg wound infections after harvesting NT-SVGs was not high. However, peripheral neuropathies, such as skin numbness and tingling, were frequently observed in the harvested side legs, which occurred up to one year. NT-SVGs could provide promising graft patency and improve clinical outcomes of CABG surgery.

**Table:** 

Authors’ Roles & Responsibilities	
HT	Substantial contributions to the conception or design of the work; or the acquisition, analysis, or interpretation of data for the work; drafting the work or revising it critically for important intellectual content; final approval of the version to be published
SS	Substantial contributions to the acquisition of data for the work; final approval of the version to be published
HK	Substantial contributions to the acquisition of data for the work; final approval of the version to be published
TW	Substantial contributions to the acquisition of data for the work; final approval of the version to be published
SS	Substantial contributions to the acquisition of data for the work; final approval of the version to be published
AT	Substantial contributions to the acquisition of data for the work; final approval of the version to be published
AS	Substantial contributions to the acquisition, analysis, or interpretation of data for the work; final approval of the version to be published
TK	Substantial contributions to the acquisition, analysis, or interpretation of data for the work; final approval of the version to be published
